# Defining Ecosystem Assets for Natural Capital Accounting

**DOI:** 10.1371/journal.pone.0164460

**Published:** 2016-11-09

**Authors:** Lars Hein, Ken Bagstad, Bram Edens, Carl Obst, Rixt de Jong, Jan Peter Lesschen

**Affiliations:** 1 Wageningen University, Wageningen, The Netherlands; 2 Wealth Accounting and Valuation of Ecosystem Services (WAVES) Program, The World Bank, Washington, DC, United States of America; 3 Geosciences & Environmental Change Science Center, U.S. Geological Survey, Denver, Colorado, United States of America; 4 Statistics Netherlands, Den Haag, The Netherlands; 5 Melbourne Sustainable Society Institute, University of Melbourne, Parkville, Australia; 6 Alterra, Wageningen UR, Wageningen, The Netherlands; Chinese Academy of Forestry, CHINA

## Abstract

In natural capital accounting, ecosystems are assets that provide ecosystem services to people. Assets can be measured using both physical and monetary units. In the international System of Environmental-Economic Accounting, ecosystem assets are generally valued on the basis of the net present value of the expected flow of ecosystem services. In this paper we argue that several additional conceptualisations of ecosystem assets are needed to understand ecosystems as assets, in support of ecosystem assessments, ecosystem accounting and ecosystem management. In particular, we define ecosystems’ capacity and capability to supply ecosystem services, as well as the potential supply of ecosystem services. Capacity relates to sustainable use levels of multiple ecosystem services, capability involves prioritising the use of one ecosystem service over a basket of services, and potential supply considers the ability of ecosystems to generate services regardless of demand for these services. We ground our definitions in the ecosystem services and accounting literature, and illustrate and compare the concepts of flow, capacity, capability, and potential supply with a range of conceptual and real-world examples drawn from case studies in Europe and North America. Our paper contributes to the development of measurement frameworks for natural capital to support environmental accounting and other assessment frameworks.

## Introduction

In recent decades, a large amount of research has been devoted to understanding the importance of ecosystems for people [1, 2]. Ecosystems are often analysed in terms of the services they provide to society, see e.g., the assessment frameworks of the Millennium Ecosystem Assessment (MA) [3], The Economics of Ecosystems and Biodiversity (TEEB) [4] and the Intergovernmental Platform on Biodiversity and Ecosystem Services (IPBES) [5]. The System of Environmental-Economic Accounts–Experimental Ecosystem Accounting (SEEA EEA) framework [6, 7, 8] examines how the contributions of ecosystems to people can be understood in terms of both services provided and in terms of ecosystems being an asset, i.e., systems that can regenerate and provide a flow of services over time depending upon their health or condition. Each framework has different potential applications, from supporting specific assessments of societal costs and benefits of ecosystem change to, as in the SEEA EEA, recording ecosystem assets and services in an accounting framework [9–11].

Although these frameworks have slightly different interpretations (compare e.g., MA and TEEB), the concept of ecosystem services is generally used to express flows of goods or services provided by ecosystems to people (via economic production or directly to individuals and society) over a specific time period. Flows to people have been labelled ‘final ecosystem services’ whereas flows of services between ecosystems are often referred to as ‘intermediate services’ or ‘intra-ecosystem flows’ [12, 7]. Our paper focusses on defining capacity and related concepts for final ecosystem services, but we revisit the relation with intermediate ecosystem services in the Discussion. In general, ecosystem service flow is a function of the ecosystem type (e.g., forests, wetlands), its biophysical setting and condition, and its accessibility and use by people. In this paper, we use condition, in line with the SEEA EEA, to indicate the state of the ecosystem. Condition indicators may express, for example, specific aspects of ecosystem structure (e.g., standing biomass, species occurrence) or processes (e.g., Net Primary Production). The actual flow of ecosystem services in a given year does not have to be sustainable, i.e., it may be that the use of an ecosystem service is greater than that which can be generated in a sustainable manner over the long term. Over time, the use of ecosystem services beyond sustainable supply levels typically leads to ecosystem depletion and/or degradation. Based on MA we interpret degradation as a change in ecosystem condition negatively affecting the ecosystem’s structure, functioning, resilience and/or ability to provide ecosystem services. Depletion is more commonly interpreted as a reduction in a specific, harvested stock, as in depleting fish or timber stocks. Degradation may involve depletion of stocks contained in the ecosystem but may also be confined to changes in processes or resilience. Both degradation and depletion reflect changes in the ecosystem asset.

To assess the status of ecosystems and their use, both the actual service flow and the flow that can be sustainably generated by ecosystems are relevant. This has been recognised in a range of studies. For example, Turner and colleagues [13] and Haines-Young and Potschin [14] refer to ecosystem function as the capacity of ecosystems to provide services. Other studies [15] describe the concept of ecosystem potential, i.e., the potential supply of services that can be generated in an ecosystem. Schröter and colleagues [16] provide a case study in which capacity is analysed for eight different ecosystem services, and Zank and colleagues [17] refer to the terms capacity and flow, relating capacity to the ability of ecosystems to supply services. However, even though several studies have explored the concept of capacity, e.g. [18], there is still no clear guidance on how the capacity of ecosystems to provide services can be defined and applied, especially when considered in the context of different categories of provisioning, regulating and cultural services. This is particularly urgent in view of the need to better understand how ecosystem assets can be defined for natural capital accounting [8].

The objective of this paper is to propose and demonstrate a set of concepts that are needed to understand ecosystems as assets in natural capital accounting. A main motivation of our work is that there is a need to better understand how ecosystem assets can be understood in monetary terms. We define ecosystem capacity as a central concept in understanding ecosystem assets for the purpose of ecosystem assessments and natural capital accounting. We postulate that, in addition to ecosystem capacity, two related concepts are important in order to understand ecosystems’ ability to generate services, and we have labelled these concepts ‘capability’ and ‘potential supply’ (the latter building upon [15, 19]and analogous to ‘theoretical supply’ described by Bagstad and colleagues [20]. In this paper, we examine how ecosystem capacity, capability and potential supply can be operationalised for the three types of ecosystem services (provisioning, regulating, cultural). We provide illustrations of the three concepts using case studies from the Netherlands, Norway and the United States. We also demonstrate that our definition of capacity allows for a meaningful conceptualisation of ecosystem degradation.

Our paper focuses on defining and testing capacity and related concepts in the context of the SEEA EEA framework [7]. The SEEA EEA has been designed to be consistent with the U.N. System of National Accounts (SNA)[21], the standard for economic statistics used by statistical agencies world-wide. In the SEEA EEA framework, ecosystem assets are measured in physical terms on the basis of extent and condition. In monetary terms, ecosystem assets could be valued based on market transactions where such transactions reflect the full value of the basket of ecosystem services (including non-market services)–however since this is unlikely to be the case in most transactions between buyers and sellers of specific ecosystems, the SEEA proposes that valuing ecosystem assets is commonly done on the basis of the net present value of the expected flow of ecosystem services [7, 8], see also the section “Analysing capacity in monetary terms.” An immediate issue is that the expected flow of services provides a specific perspective of viewing ecosystem assets, i.e., one based on current patterns of use. It may well be that the ecosystem asset has a different value under a different pattern of use. This paper demonstrates the differences between the two perspectives and analyses the implications of these differences for ecosystem accounting and ecosystem services assessment. The central premise of this paper is that several different concepts are needed to fully understand ecosystem assets in both physical and monetary terms. The application of such concepts depends upon the context of the analysis. Thus, while specifying these concepts is relevant for SEEA ecosystem accounting, we believe it is also relevant for other assessment frameworks such as those developed for TEEB and IPBES.

## Defining Ecosystem Capacity, Capability, Potential Supply, and Flow

### Defining the concepts

Ecosystem capacity builds upon the concept of ecosystem functions [22, 13]. Ecosystem functions have been identified as ecological properties that underlie the supply of ecosystem services, visualized for instance in cascade diagrams [14]. However, few studies have managed to quantify ecosystem functions and a systematic framework for defining and measuring ecosystem functions has never been developed. In the SEEA EEA framework [7], capacity is a function of ecosystem condition and extent, and it is related to expected service provision (paragraphs 2.36, 4.24 and 4.25 in [7]) and (maximum) sustainable yield (paragraphs 2.37, 2.96). Nevertheless, a clear definition of capacity is not provided in the SEEA EEA framework. Recent experiences with ecosystem accounting [23–26], and also the recent Technical Recommendations for SEEA EEA [8] show that there is a need to better define the concept of capacity and related concepts and how they can be applied to the different types of services. We first focus on capacity, and subsequently analyse two related concepts, i.e., the potential supply of ecosystem services, and ecosystems’ capability to generate those services. We contrast these definitions with ecosystem service flow, using the definition for ecosystem services from the SEEA EEA framework.

We use three general considerations to guide our effort to more precisely define capacity. First, capacity needs to be analysed for specific ecosystem services. The capacity of a forest to supply, say, timber will be different from its capacity to supply non-timber forest products or regulate water flows. In other words, capacity must reflect the stock of ecosystem capital or an ecosystem asset and its ability to supply individual services as a flow over time. Second, even though capacity needs to be related to specific ecosystem services, in practice a single ecosystem asset provides a basket of ecosystem services, and the capacities for each of these services are interlinked. For instance, a high capacity to generate timber would typically be negatively correlated with the same ecosystem’s capacity to support tourism or capture air pollutants because the supply of these services would be reduced when timber harvesting is increased. Third, we aim to define capacity in such a way that it is relevant for understanding ecosystems as assets, meaning that it must be possible to quantify capacity in both physical and monetary terms.

Given these considerations, we define **capacity** for individual ecosystem services as ‘*The ability of an ecosystem to generate a service under current ecosystem condition and uses*, *at the highest yield or use level that does not negatively affect the future supply of the same or other ecosystem services from that ecosystem*.’ ‘Current ecosystem condition’ means that the capacity is measured for an ecosystem ‘as it is now,’ *i*.*e*., not in relation to what its condition might be under alternative situations. This implies that capacity is defined independently from normative or historical baseline or reference conditions. ‘Under current uses’ means that capacity considers the type of use or management regime currently in place for an ecosystem, which would also reflect the supply of a specific basket of ecosystem services. For instance, timber extraction may not be possible where forest stands are on steep slopes, in remote and inaccessible areas, or in a natural park where logging is prohibited. In cases where the ecosystem service of ‘timber harvesting’ is limited by physical or institutional factors, capacity for this service must be assumed to be lower in view of these use restrictions, and it would be zero where no timber harvesting is currently possible.

We recognize that ecosystem services supply only materializes when there is demand for the service (note that consistent with the measurement principles of the SNA we equate ‘supply’ and ‘use’ of the service). For example, wetlands and riparian vegetation in remote parts of Siberia or Arctic Canada may mitigate floods, but if no beneficiaries live downstream, a service is not provided. In our definition we postulate that a capacity assessment needs to consider demand for the service. In the absence of a demand for the service, there is no exchange value for the service and both service and capacity do not exist. This formalisation means that there can be no capacity without a service, and that capacity is a concept that has both physical and monetary relevance. This definition and clarification above is aligned with and required for application in the SEEA EEA framework (paragraphs 4.1, 2.36, 2.37, 2.96, 4.24 and 4.25).

When all ecosystem services are used at a level below or equal to capacity, it is implied that the supply of services is, in theory, sustainable in perpetuity. In general, ecosystem services supplied at a level above an ecosystem’s capacity would lead to a degradation of the ecosystem, as reflected in the various ecosystem condition indicators. Given that any removal of materials from an ecosystem is likely to affect ecosystem structure and/or functioning in one way or another [27], a crucial qualification is that degradation represents a sustained, substantial decline in ecosystem condition at a time frame of several years or more. Both the ‘sustained’ and ‘substantial’ aspects of service decline are somewhat open to interpretation based on the context of the analysis. In this context, ecosystem use that would lead to disturbances from which the ecosystem fully recovers within a few years would not be considered to represent ecosystem degradation and would therefore not be considered to ‘negatively affect the future supply of the same or other ecosystem services’ definition of capacity. This definition is most appropriate at aggregated scales, e.g., of the landscape and above. If capacity is assessed over too small an area, degradation may be overstated because natural fluctuations will more strongly influence the ecosystem’s state relative to when larger areas are assessed. For instance, in ecosystems that are naturally subject to wildfire, small-scale analysis would show mosaics of degrading and recovering ecosystems, while at aggregated scales this would only show up if the area being burned was increasing over time. Shifting cultivation, which is often considered to be a sustainable ecosystem management strategy at very low population densities (< 1 person km^2^), is another example.

In some cases, it may be relevant to assess ecosystems’ ability to generate services irrespective of the demand for those services [17, 28]. For instance demand for a service might grow in the future (e.g., if population densities in remote, sparsely populated areas were to increase in the future). In other cases, it may be relevant to model supply irrespective of demand as an intermediate step in the development of ecosystem services models. We propose that, in line with other studies [17, 28] ecosystems’ ability to generate services irrespective of demand for such services is labelled **potential supply.** As with capacity, potential supply also requires the supply to be sustainable, i.e., there should not be a reduction in the ability to supply the ecosystem service under consideration, or other services, when the ecosystem service is supplied at the potential level. Potential could be analysed with or without consideration of legal or institutional restrictions to ecosystem use. If potential is analysed as an intermediate step in a modelling exercise, consideration of such restrictions may not be important. However, if it is used to indicate ecosystem use under changing socio-economic conditions (e.g., a growing population) then such restrictions may be important to consider.

In addition to capacity and potential supply, it may also be relevant to understand which services could be provided by an ecosystem if it were managed differently. For instance, suppose users decided to increase the use of a specific service compared to current levels (e.g., to increase timber or fish extraction). This increase in harvest may remain below or at a sustainable harvest level, but there is also still a negative effect on the supply of other ecosystem services [29, 30], which makes it different from capacity. We define **capability** as “an ecosystem’s *ability to sustainably generate one ecosystem service under current condition and type of use*, *and irrespective of potential impacts of increasing supply on the supply of other ecosystem services*.*”* Capability requires there to be a demand, since increasing the supply of a specific service is only meaningful if there is a demand for that service. Type of use reflects that currrent legal and institutional use restrictions and ecosystem management (e.g., production forest versus strict protected area) need to be considered when capability is quantified, analogous to the conceptualisation of capacity. Capabilities for individual ecosystem services cannot be summed because increasing the supply of one or more ecosystem services above the level of capacity may come at the expense of the supply of other services. For example, if pasture production in managed pastures is increased by sowing enhanced pasture species, this may affect grassland bird species diversity and hence recreational opportunities for bird watching.

An important restriction that applies to capability, capacity and potential supply is that they are assessed under current land cover and ecosystem use and composition. Changing the landscape to a different production environment (e.g., replacing natural forests by plantations, in this case a change in both the ecosystem and its management) would also change ecosystem services supply, but this is not considered in analysing capability, capacity, or potential supply, i.e., these concepts are analysed for the landscape or accounting area under its current ecosystem type.

We formally define the concepts of flow, capacity, potential supply, and capability in Table 1. The four concepts can also be illustrated with a simple example. First, consider the harvest of blueberries in a Norwegian forest. Imagine that the forests in a given municipality generate 6,000 kg of blueberries in the year 2016. Of this 6,000 kg, 3,000 kg are consumed by wildlife including moose that are hunted for meat, and 1,000 kg are harvested by people. The remaining 2,000 kg are not consumed because demand for these berries from either animals or people is already satiated. In this case, the flow of the service is 1,000 kg as this is the actual harvest. the potential supply is 3,000 kg (i.e., what is available for human use without affecting the supply of other ecosystem services such as moose hunting). Capacity and capability are also 1,000 kg since only the amount for which there is demand is included and this amount can be harvested without compromising future supply of blueberries (relevant for capability) as well as without compromising the future supply of other ecosystem services (relevant for measuring capacity). Now imagine a blueberry jam factory is opened nearby and the demand for, and subsequent harvest of blueberries (i.e., the flow) increases to 5,000 kg blueberries per year. The potential supply remains 3,000 (i.e., the amount that can be supplied on a sustainable basis, irrespective of demand) and the capacity increases also to 3,000 kg because harvests at this level will not affect the future flow of blueberries or other ecosystem services (note that there is demand for this amount of blueberries). The capability increases to 5,000 (i.e., this can be harvested without disrupting the flow of blueberries over time, and there is actual demand for this amount of berries; a possible negative effect on moose populations due to food shortages is accepted). In both situations, the four concepts can be conceptualised, in principle be quantified, and are meaningful for ecosystem management.

**Table 1:** Ecosystem service capacity, capability, supply, and flow concepts.

Concept	Formalisation
Ecosystem service flow	= f(E_t_, C_t_, M_t_ | D)
Capacity	= f(E_t_, C_t_, M_t_ | D, S)
Potential supply	= f(E_t_, C_t_, M_t_ | S)
Capability	= f(E_t_, C_o_, M_o_ | D, S)

Note: E_t_ = ecosystem extent at present (in year t); C_t_ = Condition at present (in year t); M_t_ = management at present (in year t); C_o_ = Under optimal conditions for the supply of a specific service; M_o_ = management required for optimal supply of a specific service; D = represents demand for the service; S = at sustainable harvest rate.

### Capacity for different types of ecosystem services

In this section, we discuss how capacity can be operationalized for the three main types of ecosystem services: provisioning, regulating and cultural services. For provisioning services, the capacity of an ecosystem to generate the service would normally depend on the (re)growth of the service-producing asset involved (e.g., timber, fish, or fodder)–with (re)growth itself usually a function of, for example, population size, ecosystem condition, and other factors. In basic biophysical models, regrowth is often assessed vis-a-vis the carrying capacity of the ecosystem for the species involved. However it is increasingly clear that defining carrying capacity itself may not always be straightforward since it varies as a function of (stochastic) variables such as ecosystem dynamics and climate variability [31]. Regrowth may also be affected by other natural and human factors that lead to losses in the stock (e.g., fire or storm damage to the timber stock, ocean pollution impacts on fisheries).

For provisioning services, actual ecosystem service flow (e.g., timber or fish harvest) in a given year may be less than, equal to, or greater than the capacity (in the latter case an ecosystem can be expected to be subject to degradation). Capacity can only be greater than actual flow in cases where an increase in the use of an ecosystem service (compared to actual harvest levels) would not lead to a sustained, substantial decline in the availability of other ecosystem services. In practice, it may only rarely be possible to increase the extraction rate of an ecosystem service without substantially reducing the ecosystems’ capacity to generate other services, particularly in intensively used ecosystems [32]. Extraction of provisioning services by definition affects ecosystem structure and functioning. Hence, the concept of capacity requires a consideration of the scale of the analysis and whether the effect is sufficiently large to materially impact ecosystems’ ability to generate other services.

Regulating services result from ecosystem processes and functioning. A flow of these services may emerge from either naturally occurring processes independent of any human intervention (e.g., carbon sequestration in natural forests) or from deliberate interventions in the ecosystem (e.g., reforestation financed by a carbon project). We assume in both cases that capacity equals flow, since the use of a regulating service does not alter the ecosystem (even though modifying the ecosystem to enhance the supply of a regulating service may do so), making the use of a regulating service in principle always sustainable. For carbon sequestration, capacity also equals potential supply since the service is global (i.e., everybody benefits from this service regardless of where the sequestration takes place). For all other (non-global) regulating services, potential supply may be equal to or higher than flow and capacity. For instance, imagine an ecosystem in an upper watershed supplying a water regulation service resulting in flood control for people living downstream, analyzed by means of rainwater infiltration. The actual flow of the service (expressed in mm per time unit) is represented by the water infiltration rate that is achieved under current rainfall conditions. The potential supply would equal the maximum infiltration rate and may be relevant in the future in case climate change would locally increase rainfall intensity–it may be equal to or higher than the present flow of the service (but cannot be lower unless there are changes in ecosystem condition). Rainwater infiltration may also occur in upper watersheds with no people living downstream that would benefit from reduced flooding. In this case there is no service being supplied, but there is still a potential supply (which may become relevant when people start settling in the downstream area) [17, 28].

For cultural services, capacity may be defined as the use level of these services that would not lead to declines in ecosystem condition. For example, this trade-off is explicitly expressed in the enabling legislation for the U.S. National Park Service, which is charged with “conserve(ing) the scenery and the natural and historic objects and the wildlife therein and to provide for the enjoyment of the same in such manner and by such means as will leave them unimpaired for the enjoyment of future generations”–a mission that has been emulated by many park management agencies around the world. Flow may exceed capacity, for instance, in the case of (i) overcrowding of tourists in a national park; or (ii) if the number of tourists or other activities related to cultural services is so high that it affects other ecosystem services generated by the park. Defining capacity for cultural services is not straightforward [33], but the different concepts can be illustrated with an example related to tourism in a national park. The flow of the service can be expressed in terms of the number of visitors received or as the number of overnight stays generated in nearby hotels or other park accommodations over the accounting period (e.g., in a year). The capacity of the service could be analysed on the basis of the maximum number of visitors that can be received without causing overcrowding or disrupting other ecosystem services, assuming that there is demand for this number of visitors. For some sensitive ecosystems with high tourism demand (e.g., the Galapagos Islands), capacity estimates may be available. Potential supply can be interpreted as a measure of the potential attractiveness and accessibility of the national park, independent of actual demand for tourism-related ecosystem services. This was calculated, for example, by Maes and colleagues [34] for European landscapes based on the type of ecosystem, using a relative scale (from 1 to 9). In this ranking, a high score was attributed to large, non-fragmented ecosystems with forest cover that are easily accessible by road. Capability would be larger than capacity in cases with high tourism demand where park managers were not concerned about, for example, impacts of mass-tourism on biodiversity or other ecosystem services. The different concepts are explained for the three types of services in Table 2.

**Table 2:** Ecosystem service flow, capacity, potential supply, and capability for provisioning, regulating, and cultural services.

Concept	Provisioning services	Regulating services	Cultural services
Flow	The amount of service extracted by people in a given time period.	The amount of service received by people in a given time period.	The amount of service received by people in a given time period.
Capacity	Sustainable harvest rate under present ecosystem condition and management, under the condition that capacity cannot exceed demand for the ecosystem service. Under current management implies that legal and institutional restrictions on ecosystem use apply.	Capacity equals flow for regulating services.	Number of activities (e.g., recreational visits) that can take place without overcrowding or damaging the ecosystem, under the condition that capacity cannot exceed demand for the ecosystem service.
Potential supply	The amount of service that can be sustainably generated by an ecosystem independent of demand for the service.	The amount of service that can be generated by an ecosystem independent of demand for the service. For carbon sequestration, capacity = potential supply.	The amount of service that can be sustainably generated by an ecosystem independent of demand for the service.
Capability	Ability to sustainably generate an ecosystem service under the current ecosystem conditions, but with ecosystem use that would prioritize the sustainable supply of this service (and accepts a decline in the capacity to generate other services). Capabilities for different ecosystem services may not be additive/compatible.	Ability to sustainably generate an ecosystem service under the current ecosystem conditions, but with ecosystem use that would prioritize the sustainable supply of this service (and accepts a decline in the capacity to generate other services).	Ability to sustainably generate an ecosystem service under the current ecosystem conditions, but with ecosystem use that would prioritize the sustainable supply of this service (and accepts a decline in the capacity to generate other services).

**Fig 1:**
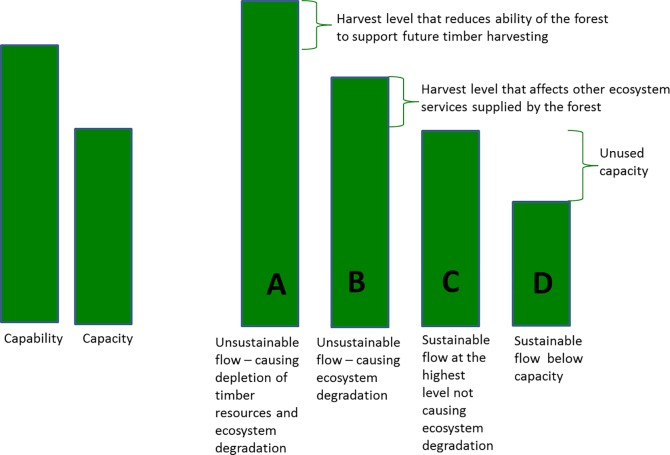
Timber harvesting capability, capacity, and four levels (A, B, C, D) of flow, in this case representing harvest. The height of the bars indicates the relative harvest levels under four management types.

The concepts of flow, capacity and capability are also explained in Fig 1 below, with the example of timber harvesting. For provisioning services, flow can be greater than, equal to, or less than capacity and capability. A flow of the service that exceeds capacity points to unsustainable use if sustainability is assumed to not involve a reduction of any ecosystem services currently supplied by an ecosystem. A flow exceeding capability can be considered unsustainable since, if the extraction rate is sustained over time, it will reduce the flow of other services as well as the harvested service itself, as illustrated below for depletion of timber resources. The figure does not show the potential supply, which can be equal to or higher than capacity (but not lower), and which can be lower than, equal to or higher than capability (see text above).

### Analysing capacity in monetary terms

In national accounting, assets can be valued on the basis of direct transactions of assets, or, when such transaction data are not available, on the basis of the Net Present Value (NPV) of the expected (capital) service flows that the asset will provide over its lifetime [21]. Ecosystem accounting must consider that ecosystems generate multiple services, including market and non-market services. In general, marketable ecosystem goods and services (e.g., where the value of soil functions, terrain, climate, etc. from croplands are embodied in crop production) will be considered in market transactions of ecosystem assets. However, non-market ecosystem services (e.g., soil carbon sequestration) will not generally be considered by the buyer or seller of an ecosystem asset. Therefore, in most cases, direct transaction values of assets are not a sufficient valuation approach for ecosystem assets. Hence, in the SEEA EEA, it is proposed that the ecosystem asset generally needs to be valued on the basis of the NPV of the expected flow of ecosystem services. This flow of ecosystem services may be supplied indefinitely where under sustainable management, or it may end when the ecosystem is entirely degraded. Clearly, changes in ecosystem management may reduce or extend the time that an ecosystem can generate services at a certain level.

Forecasting ecosystem service flows is a challenge in analysing ecosystem asset value on the basis of the NPV of the expected service flow, particularly when these flows are non-sustainable. Ecosystems often do not change in a predictable, linear fashion, but may have complex dynamics such as multiple steady states, thresholds and hysteresis, as a function of positive and negative feedback mechanisms guiding ecosystem dynamics. A well-known example is the Newfoundland cod fishery, which unexpectedly collapsed in 1982 following a sustained period of overfishing. In spite of strict fishing regulations afterwards, fish stocks have not returned to pre-collapse levels [35]. Estimating ecosystem asset values taking into account future changes in ecosystem service flows due to unsustainable management is likely to remain a key challenge in ecosystem accounting in the foreseeable future, and must be informed by a scientific understanding of ecosystems’ underlying ecology.

An alternative way of conceptualising the value of an ecosystem asset is based on capacity, expressing the value of an ecosystem in its current conditions under sustainable management. This indicates ecosystems’ value under sustainable use, subject to existing demand for their services. As it does not require quantification of future service flows, this is often more straightforward from a modelling perspective (values of ecosystem services, of course, may still change over time). However, a valuation based on capacity should be seen as an additional indicator because this valuation is not consistent with the general concept of exchange value, which underpins the SNA’s asset value definitions [21]. This inconsistency means that capacity based valuation cannot be directly compared or integrated with the valuation of other assets already included in the national accounts balance sheet (e.g., buildings or machines).

The capacity value indicator is, however, relevant for ecosystem management in the sense that it estimates ecosystems’ prospective value under sustainable management that can, in theory, be maintained infinitely. The difference between the two indicators, i.e., between the asset as currently managed and the asset as sustainably managed, is also of interest. For unsustainably managed ecosystems, this difference can be seen as the cost (or the benefit, in terms of short-term economic returns of unsustainable extraction) of unsustainable management.

The asset value under current management can be less than, equal to, or greater than the asset value under sustainable management. This will depend upon several factors, such as the relationships between ecosystem services (co-benefits or in competition), the actual condition, as well as the discount rate used, in both cases, to compare future and present benefits. The SNA [21] specifies that market discount rates should be used in quantifying NPV. Whether this is also appropriate for non-market ecosystem services in ecosystem accounting remains an open question [36]. Transactions in the SNA are considered on the basis of exchange values under which willing and informed buyers and sellers are prepared to exchange goods in a market of sufficient size to enable establishment of a market price. Likewise, the discount rate should reflect market transactions, i.e., be based on discount rates applied in the money market. However, the market clearly does not establish discount rates for non-market ecosystem services such as air filtration or carbon sequestration (in spite of carbon markets where prices are determined to a large degree by market design). Hence, it may be appropriate to value non-market services using a public discount rate in ecosystem accounting, pending further discussion in the academic and statistical fields.

**Fig 2:**
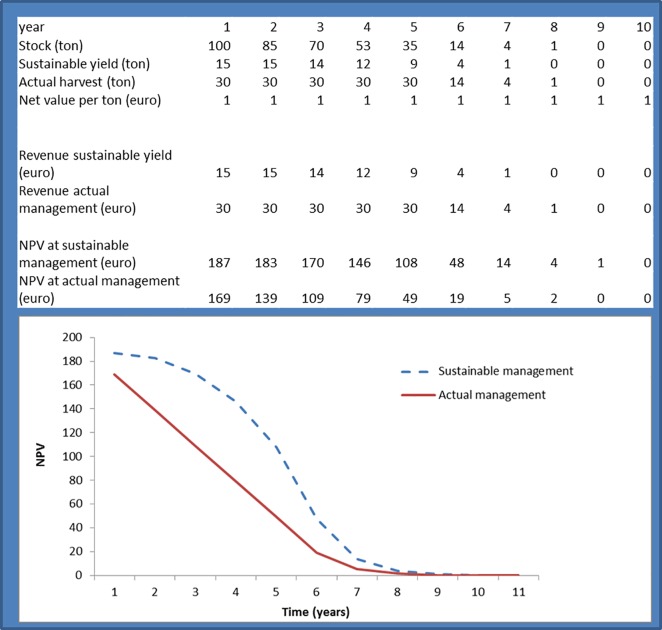
Valuing capacity: a quantitative example for a hypothetical ecosystem supplying one provisioning service. NPV: Net present value.

The difference between NPV based on expected service flow and sustainable flows (i.e., capacity) is presented in Fig 2. The example presented uses a stylised ecosystem model providing only one provisioning service that can be harvested at different rates. In any given year, the ecosystem manager could adopt a sustainable harvest regime, and the NPV generated by sustainable management as of that year is also indicated in the table. Regeneration of the resource is on the basis of a simple logistic growth curve, with a carrying capacity of 200 tons and a logistic growth factor of 0.3. For the purpose of simplicity, a 5% discount rate and 20-year discounting period are used. There are several simplifications in the model, including the assumption of constant net revenue values (in reality both prices and harvesting costs may go up with increasing scarcity). It is clear that both the absolute asset value and changes in asset value differ for each method. It should be stressed that the obtained values depend on the chosen model and various assumptions such as the regeneration rate of the ecosystem, the rate of overharvesting, prices as well as the choice of the discount rate and discounting period. It is equally possible to construct an example in which the value based on expected service flow lies above that of the sustainable flows.

When ecosystem degradation is assessed based upon the change in value of the asset that occurs during the accounting period, it is clear that these two approaches lead to two alternative measures of the cost of ecosystem degradation. As will be discussed in the section “Challenges in operationalising capacity, capability and potential”, the valuation of ecosystem degradation is a complex issue, and further research in this area is needed.

## Case Studies

### Introduction

In this section we provide three quantitative real-world illustrations of flow, capacity, potential supply, and capability, drawing on earlier studies. These include Limburg province in the Netherlands, Telemark County, Norway and the Puget Sound in the U.S. Pacific Northwest. Below we summarize for each area the implications for quantifying capacity, capability, and potential supply of a regulating service (carbon sequestration, Limburg), a provisioning service (timber harvest, Telemark) and a cultural service (scenic viewsheds, Puget Sound). We identify indicators and provide maps for selected concepts. More details on these case studies are provided elsewhere [16, 17, 26, 28]. All are relatively data-rich environments, and we acknowledge the need for a significant amount of data in order to model these concepts for a comprehensive set of ecosystem services.

### Comparing flows, capacity and capability for carbon sequestration in Limburg, Netherlands

#### Case study area

Limburg province is situated in the south-eastern part of the Netherlands and covers some 2,200 km^2^. Unlike the rest of the country, the southern part of the province is hilly, and the province is known nationally for its attractive landscape. Limburg is dissected by the river Meuse, which flows from south to north and is bordered on both sides by several Quaternary fluvial terraces. The province has a mix of forest and agricultural landscapes, influenced by human management since at least Roman times. The area is densely populated (522 people/km^2^ in 2012) and competition for land for agricultural, nature and urban purposes is high.

#### Methodology

Seven ecosystem services were analysed for the whole province in both physical and monetary terms in earlier studies [24,37]. Here we build upon this earlier work but expand it to show how capability can be quantified for ecosystem accounting, with a focus on carbon sequestration. For this service, as explained above, flow, capacity, and potential supply are all equal (given that carbon sequestered anywhere on the planet is of equal societal importance). Capability, however, is different, since with specific ecosystem management measures, carbon sequestration can be enhanced. We show for crops and grasslands the amount of carbon that is annually sequestered in the soil with current management (i.e., flow, capacity and potential supply of the service) and with enhanced management (capability). Results are based on Lesschen et al.[38], who assessed the soil organic carbon (SOC) sequestration possibilities for seven agricultural management strategies in the Netherlands. Here we show the maximum possible SOC sequestration potential for zero tillage and the realistic possibility for a combination of measures (reduced tillage, zero tillage, catch crops, improved crop rotation, incorporation of crop residues in the soil, management of field edges and reduced grassland renovation). Zero tillage involves the use of adapted machinery to direct sow in the stubble, which reduces the degradation of organic matter.

To quantify the level of carbon sequestration that could result from these management strategies, the MITERRA-NL accounting model was used. This model assesses the effects and interactions of policies and measures in agriculture on greenhouse gas emissions on a regional scale using the SOC stock change approach of the Intergovernmental Panel on Climate Change (IPCC) 2006 guidelines [39] in combination with country-specific SOC stocks derived from the Dutch soil profile database [40]. The current SOC sequestration rate (the ecosystem service flow) is calculated with the RothC soil carbon model [41]. The annual SOC sequestration rate from the different management strategies is calculated by dividing the total SOC stock change by the 20-year equilibrium period following the IPCC 2006 guidelines. Calculations were done at the 4-digit postal code level, and resulting maps were overlain with a mask indicating crop and grasslands based on the Netherlands Ecosystem extent map [26].

#### Results and discussion

**Fig 3:**
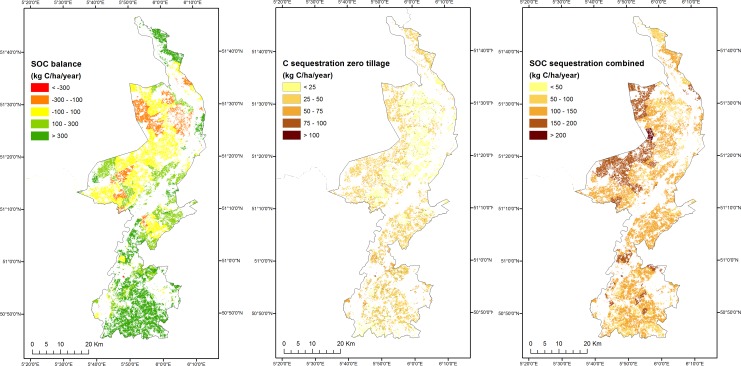
Soil organic carbon (SOC) sequestration: flow, capability when only zero tillage is applied and capability using a combination of six measures for agricultural land in Limburg, Netherlands.

The current and possible SOC sequestration rate on agricultural land under several management measures are shown in Fig 3. The current SOC balance in Limburg is positive in most agricultural areas, indicating net SOC sequestration. Positive SOC sequestration mainly occurs in grasslands, but in arable land negative SOC balances are also found. The possible SOC sequestration for the zero tillage measure is greatest, although in practice not all farmers can or will convert to zero tillage systems. The average SOC sequestration rate for a realistic scenario with a combination of several measures is 100–150 kg C/ha/year. The analysis shows that, for SOC sequestration in agricultural land in Limburg, current flow is less than capability, indicating a possibility of storing more carbon below-ground in croplands with the introduction of specific management measures. The difference between flow and capability can guide policy making aimed at enhancing carbon sequestration in agricultural land by implementing differnent management practices. These practices would not necessarily restrict crop or fodder production, for example zero tillage may improve soil structure and enhance crop growth. The case study illustrates that different management practices that enhance ecosystems’ ability to generate services may not necessarily be additive, i.e., it may not always be possible to combine them, or the sum of their combined effects may be lesser (or greater) than the sum of the effects of individual measures. The possible effect of the various measures on other ecosystem services, or their cost benefit ratio, are relevant for decision making but were not assessed in our case study, as further discussed in the section “The policy relevance of capacity and related indicators.”

### Comparing flows and capacity for timber harvest in Telemark, Norway

#### Case study area

Telemark County is located in southern Norway. It covers around 15,300 km^2^ and has a population of about 170,000 people. The county has a very diverse landscape including parts of the Hardangervida plateau as well as several mountains exceeding 1,500 m in elevation. The high-altitude alpine areas have extensive snow cover in winter and have sparse vegetation mostly consisting of dwarf trees, shrubs, herbs and mosses. The lower altitude coastal area has a more moderate climate, with vegetation consisting of pine, spruce and birch forests. Annual rainfall varies from 800 to 1,000 mm depending upon altitude and location. Agriculture is concentrated on alluvial deposits along the rivers flowing through the county, and seasonal reindeer herding and livestock grazing (mostly sheep) takes place in the uplands.

#### Methodology

Nine ecosystem services, and the capacity of ecosystems to provide these services, were assessed[16]. For this paper, we re-evaluate the ‘timber supply’ service, comparing the flow of timber and the capacity of the county’s ecosystems to support this service. Timber flow (harvested timber in m^3^/ha for the year 2010) was taken from national harvest statistics, where the finest available resolution was the municipality level [42]. It was assumed that both logs and firewood are harvested, with the assumption that the extraction level for firewood remained at its 2005 level, the last year of data collection. The county has 18 municipalities, and for each, the timber harvest was spatially allocated to forested land cover types [16]. Capacity was modelled on the basis of the national land resources dataset, which includes the whole of Telemark under the treeline [16]. Site quality classes, which are classifications of an area’s capacity to produce timber, ranged from unsuitable, i.e., <1 m^3^/ha/yr to very high, i.e.>10 m^3^/ha/yr. This spatial information was combined with annual timber regrowth statistics (m^3^/ha/yr) for the region (including Telemark and two nearby counties) taken from the most recent national forest inventory (2005–2009)[43],).

#### Results and discussion

**Fig 4:**
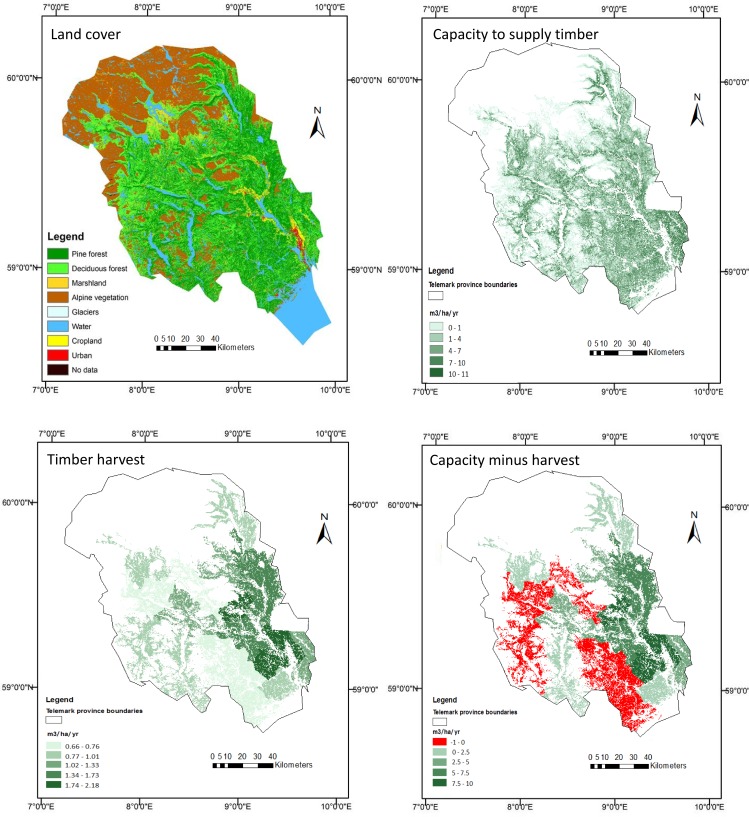
Timber harvest capacity, flow (i.e. timber harvest) and difference between capacity and flow in Telemark, Norway. Source: adapted from [15].

Most timber is harvested from areas mapped as dense coniferous and open mixed forests. Harvest included both native pine and introduced spruce wood. Fig 4 shows the flow of the timber provision service, its capacity, and the difference between flow and capacity. Information on timber harvest is only available at the scale of the municipality. As in other parts of the world, timber harvesting takes place in rotations, typically of three to four decades in Telemark. Hence, if timber harvesting is mapped on an annual basis, and capacity and flow are compared on an annual basis, it would show degradation in large areas where capacity exceeds flow, and concentrated areas (where harvesting takes place) where flow exceeds capacity. We believe a more landscape-scale approach is more useful to distribute the harvest over the concessions (as in [44]) or, in the absence of concession-specific data, by administrative zone (as we have done for Telemark).

Our analysis shows that timber harvesting is well below capacity in most of the case study area. However, in five municipalities (together covering around 40% of Telemark) flow exceeds capacity–albeit with a small difference (in each of these municipalities flow exceeded capacity by less than 1 m^3^/ha/year). Since the difference is small, and since data are available only for one year (2011), we cannot conclude that this points to unsustainable harvesting practices. A further consideration is that we allocated harvest data over administrative zones, which includes areas that are not suitable for harvesting because they are on steep slopes or too far from access roads to make harvesting economical. We also did not have information on zones were harvesting was not taking place, e.g., because of a protected status. Therefore, the actual flow in the logged area is greater than the average flow shown in Fig 4. Further research covering multiple years or the construction of an ecosystem account covering multiple years would be needed to determine whether harvest rates exceed capacity in parts of Telemark. Also, both forest stands and timber production in Telemark are dominated by only two species, pine and spruce. This approach would be more challenging to apply in ecosystems with much greater tree species diversity. In these areas, it will be important to consider both annual increment (i.e., capacity) and flow of timber for multiple different tree species, requiring more elaborate ecological data to produce the accounts.

### Comparing potential supply and flows for scenic views in the Puget Sound, U.S. Pacific Northwest

#### Case study area

The Puget Sound in the U.S. Pacific Northwest is a large (35,510 km^2^) watershed with a population of 4.4 million people including the cities of Seattle and Tacoma [17, 28]. Puget Sound is a glacially carved inlet of the Pacific Ocean, and is the second largest estuary in the United States. The watershed is bordered by the Olympic Peninsula to the west, which rises to elevations as high as 2,400 m, and the Cascade Mountains to the east, with a maximum elevation of nearly 4,400 m. The Puget Sound’s upper watersheds remain largely in coniferous forest cover, while agricultural land use occurs in the valleys and developed land mostly at lower elevations closer to the Sound.

#### Methodology

In a past study, potential supply and flows of five ecosystem services were mapped for the Puget Sound, and the percentage of each potential service that was actually used by beneficiaries was quantified when accounting for locations where services were used and service-specific flow mechanisms [28]. Here we evaluate potential supply and flows of scenic beauty from visually valued objects (e.g., mountains and water bodies) to homeowners [45]. We used the Artificial Intelligence for Ecosystem Services (ARIES)[46]) modelling platform to quantify potential supply and flows of this service. The value of scenic views typically accrues to property values and can be measured using hedonic analysis, or in this case mapped by identifying: (1) ecosystems providing high-quality views, (2) features that impede or degrade views, and (3) housing locations. Sources of high-quality views and features that degrade views were quantified using a spatially explicit Bayesian network that ranked potential view quality from 0–100 [28]. We used a review of the hedonic valuation literature [45], which ranked the influence of different viewshed characteristics on property values, to inform model development and to parameterize the models. Within a given viewshed, our models quantified the contribution of viewshed features such as mountains and water bodies to high-quality views (i.e., potential supply) and those detract from view quality, including obstructions or visual blight such as industrial or commercial development. These locations were linked by a model that computed visibility along lines of sight from use locations to scenic viewshed features, estimating ecosystem service flows. The model includes a distance decay function that accounts for changes with distance in the quality of views.

#### Results and discussion

**Fig 5:**
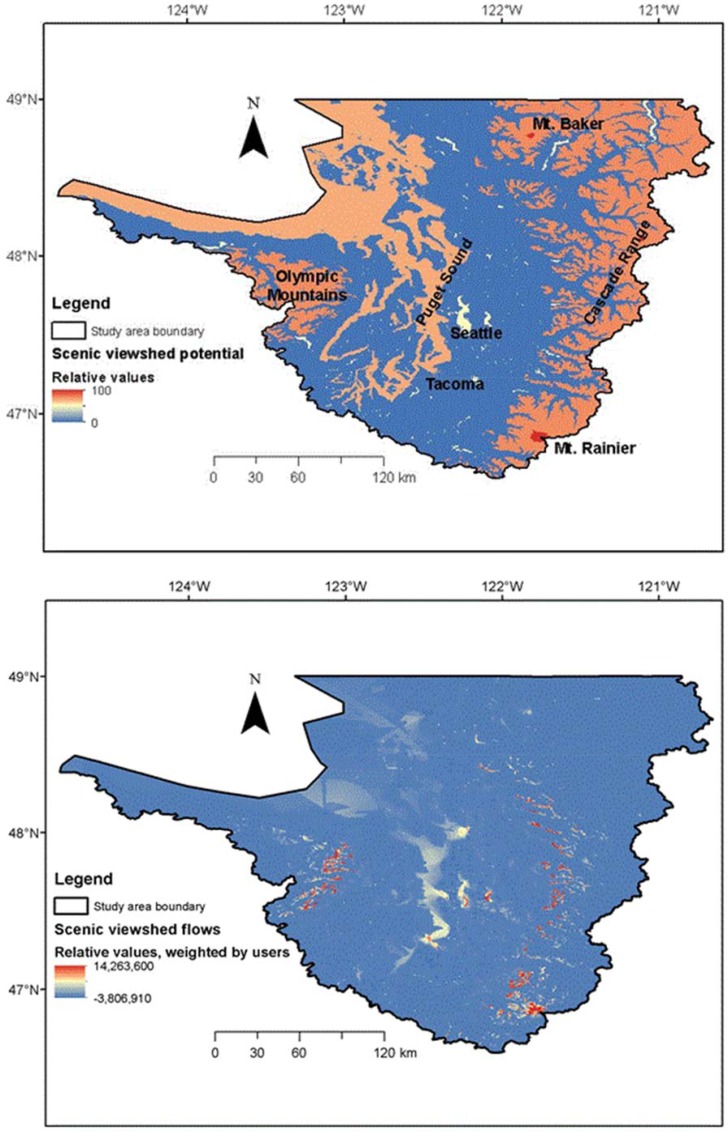
Scenic viewsheds for homeowners–potential supply and flow in the Puget Sound, U.S. Pacific Northwest. Negative values for viewshed flows occur when viewsheds intersect features that degrade view quality (e.g., commercial or industrial development). Source: adapted from [28].

When combined with spatial data for elevation and land cover, a Bayesian model of visually valued objects mapped the peaks of the Cascade and Olympic Mountains (highlighted by the tallest peaks like Mt. Rainier and Mt. Baker), followed by the waters of Puget Sound and the Pacific Ocean and inland water bodies as the most visually valued features in our scenic viewshed potential supply map (Fig 5). However, when the location of homeowners was mapped and visual connections modelled, only 15.7% of the total potential scenic views is accessible to homeowners as ecosystem service flows [28]. This is primarily because while the front of the Olympic and Cascade ranges are highly visible to a large number of beneficiaries living in Seattle, Tacoma, and other cities, the more eastward peaks of the Cascades and westward peaks of the Olympics are visible to far fewer homeowners. Bagstad and colleagues [28] also mapped the most visually valuable objects, when accounting for the number of homeowners with accessible views of such mountain and water features (their Fig 4B). As more beneficiaries, who are often more dispersed, are added to the region through population growth, the ecosystem service flow would increase, though this increase in viewshed capability is likely to cause ecosystem degradation that reduces ecosystems’ ability to produce other services. By quantifying potential supply and flows, planners can better understand how siting development or conservation for urban development can increase or decrease service flows [17].

## Discussion

### Connecting capacity and related concepts to the SEEA framework

An important catalyst for this paper has been the work program led by the international statistical community to develop environmental-economic accounting standards and guidelines. This work recently led to the development of the SEEA Experimental Ecosystem Accounting [7], which was endorsed by the U.N. Statistical Commission in 2013. In this context, the concept of capacity was discussed and recognised as a key link between ecosystem service delivery and ecosystem assets’ condition and functioning. However, no clear agreement could be reached at that time on a precise definition of capacity, nor on how the concept could be recorded in an accounting context.

We believe that our framework, illustrations, and case studies show how capacity and related concepts can be defined in a way that is aligned with the accounting principles of the SEEA EEA, is quantifiable provided that data and models are available, and can inform environmental management decisions. Our case studies illustrate that every service requires a different assessment methodology ([16, 28]. Generally the same indicators (e.g., m^3^ of product/ha/year) can be used for flow, capacity and capability, which makes it possible to compare the concepts both in terms of maps and tables. Clearly, broader testing of the concepts of capacity, potential supply, and capability is required before a more final evaluation of their applicability can be provided.

Importantly, as illustrated in Fig 2, our framework allows monetization of capacity, which is important in the development of a consistent natural capital accounting framework. We believe that capacity is an important indicator for sustainability, given that it expresses the income that can be generated over long time frames from human activities in and depending upon ecosystems. Put simply, declines in capacity would indicate that our current use patterns carry longer-term costs, while increases in capacity would suggest that our ability to manage the ecosystem service delivery to future generations is improving. The assessment of capacity as an accounting concept thus represents an addition to the suite of concepts that are relevant in the assessment of sustainability, such as thresholds and resilience [47, 48]. Potential supply and capability have not yet been discussed in the context of SEEA and may not necessarily be required core SEEA indicators. Yet these can nevertheless be important for modelling ecosystem responses to management, and for organising data on natural capital and ecosystem services.

Compared to earlier work, we have defined capacity for regulating services differently from Schröter and colleagues [16] and Zank and colleagues [17]. Ecosystems’ ability to control snow slides, for instance, was labelled capacity [16], but we now propose to label this service potential supply (i.e., supply that would occur even in the absence of demand from people) in line with [15]. The same logic would apply for services such as erosion and sedimentation control or flood regulation–flow equals capacity for these services, and flow that would occur in the absence of people is labelled potential supply. We believe that this new framework is more internally consistent compared to alternative interpretations of these concepts [15, 16, 17].

### Challenges in operationalising capacity, capability and potential supply

The SEEA EEA and other assessment frameworks consider ecosystems both in terms of flows of services and stocks of ecosystem assets or capital. While there is ample experience with defining and quantifying ecosystem service flows, this paper makes the case that a more complete understanding of ecosystem assets requires consideration of the concepts of capacity, capability and potential supply, in addition to indicators of ecosystem extent, condition and the net present value of the expected flow of ecosystem services. This will generally require the application of a range of different ecological and bio-economic models in order to spatially and temporally analyze the various condition, asset and service flow indicators. An interesting type of model in this context is the portfolio decision model [49] that allow the integrated analysis of a range of relevant variables. Among other modeling platforms ARIES may be particularly useful given its flexibility and conceptualisation of ecosystem services that generally aligns well with the accounting approach [46]. While not all assessments would necessarily require quantification of all of these concepts, a clear understanding of the different concepts is important in ecosystem services research. In the further testing of the concepts of capacity, potential supply, and capability, we see the following challenges and opportunities:

### Considering capacity, potential supply, and capability in the context of final and intermediate services

Our paper has only considered final ecosystem services. The recent SEEA EEA Technical Recommendations, as well as various other papers [12] also recognise ‘intermediate services,’ reflecting interactions between ecosystems. In considering intermediate services, care needs to be taken that double counting (for example with related final services) is avoided. Information on intermediate services can be highly relevant for environmental management [7]. Often, intermediate services underpin the provision of final ecosystem services, as in the case of upstream salmon spawning grounds that enable their downstream and oceanic harvest. The terms that we defined in this paper are also relevant for intermediate services. For example, in the case of extracting water for irrigation, the river ecosystem may have a certain capacity to allow extraction of water for irrigation without compromising the supply of other ecosystem services by riverine and riparian ecosystems. In this example, capacity and flow may well be different (see also [50].

Sustainable use of intermediate services implies that ecosystems can continue to provide these services to themselves–thus underpinning the final services that humans value directly and that are tracked in the ecosystem accounts. This conceptualisation aligns with the concept of “accessible surplus” [30], i.e., the flows from the environment to the economy after the environment has sufficient resources to sustain itself. These flows can be highly relevant for environmental policy. For example, in water policy terms in Australia (as in many other countries) there is much discussion of the level of “environmental flows”–i.e., identifying the balance between abstracting water for irrigation and the quantity of water that should remain in the river/catchment to maintain a chosen level of river condition [50]. In drought conditions such discussions are crucial for finding a balance between satisfying short term needs of farmers and other water users and ensuring longer term functioning of the ecosystem. Further work, however, is needed to assess if the concepts of capacity, capability, and potential supply are applicable and useful to apply in the context of analysing intermediate services.

### Integrating degradation into natural capital accounts

The measurement and valuation of degradation has been debated since the first discussions of environmental accounting [51–53]. In this paper we show that ecosystem degradation can be valued as both in change in the value of the expected flow of ecosystem services and in the value of a change in capacity that occurs during the accounting period as a result of human activities. The approaches represent two different perspectives on ecosystem degradation. While they provide a conceptual underpinning of degradation from an environmental accounting perspective, two remaining issues require further scrutiny.

First, capacity, potential supply, and capability can change over time (i.e., from one ecosystem accounting period to the next) due to changes in ecosystem condition and/or management choices. But natural events (e.g., storm damage) may also lead to changes in capacity, and most ecosystems will normally be subject to changes in capacity over time due to natural variability (e.g., annual or seasonal rainfall variability in dryland ecosystems) or ecological processes (e.g., succession) [54]. Changes can be labelled as natural or human-induced, though in practice it may sometimes be difficult to distinguish between human-induced and natural causes of degradation, particularly under climate change. In ecosystem accounting, this distinction is important [7] due to the requirement to align with definitions of income from which degradation costs are deducted. Naturally induced changes in capacity would be recorded in an asset account as other changes in volume, and only human-induced changes would be recorded as degradation costs. However in other assessments, analysts might choose not to make this distinction.

Second, degradation is not simply the change in the NPV of expected or sustainable flows of the ecosystem asset. Rather, the total change in NPV can be decomposed into changes due to human activity, changes due to price variability (revaluation), and changes due to natural processes and events [55]. The intent in accounting terms is to define degradation in such a way that the cost may be deducted from income earned as a result of ecosystem use. In theory, degradation costs may be attributed to the actor responsible for the decline in capacity (e.g., landowner, industry, or government). This proposed approach to defining and measuring degradation is aligned with accounting approaches developed for measuring depreciation in the context of produced assets such as buildings and machines. However, in practice it may sometimes be difficult to attribute degradation to specific actors given the long time period for negative impacts to surface and/or the cumulative nature of degradation (the issue of so-called ecological debt), and/or the existence of ecological thresholds.

### Modelling ecosystem dynamics to assess ecosystem service flows, capacity, capability, and potential supply

Accounting for natural capital assets requires a dynamic approach to analyze the net present value of flows of ecosystem services. In the case of the SEEA EEA, the asset value is generally related to the NPV of the expected flow of services, and this flow will change over time as a function of changes in ecosystem condition (e.g., depletion of species stocks) or changes in management [56]. There are two basic approaches for relating ecosystem service supply to ecosystem condition. The first approach is to examine trends in ecosystem functioning and relating ecosystem use and ecosystem change in a (spatial) statistical manner. An example is provided in Brookhuis asnd Hein[57] where the relationship between deforestation and flood prevention is examined using a spatial regression model. The second approach involves proces-based modelling, which requires capturing key ecological processes and ecosystem components in sets of differential equations [58]. An example is provided in Costanza and colleagues [59] where land use and ecosystem dynamics are linked to ecosystem services supply in a spatial, dynamic systems model. Even though a large number of such studies have been published related to a broad variety of ecosystems, there are nevertheless important data gaps [60]. These models will also be prone to substantial uncertainty, since they involve scaling up local results to the scale at which the accounts are developed, which may often be national. This topic requires the ecosystem accounting community to reach out to the work of, in particular, the Resilience Alliance, a research community that has been concerned with analysing complex ecosystem dynamics (including such aspects as multiple steady states and resilience for natural and man-induced disturbances) at multiple spatial and temporal scales [61].

### Understanding uncertainties and sensitivities

In accounting, it is important to understand the robustness of the data included in the accounts. In addition, when the accounts are used to underpin decision making on natural resource use, the decision makers should be informed of the uncertainties in the numbers. The national accounts do not by default report uncertainties in the numbers they include, even though data quality assurance and verification are central to their production. Given that ecosystem accounting is a recent development, and that there will be substantial uncertainty in the spatial and temporal models and valuation estimates required to fill the ecosystem accounts, explicit attention is needed for uncertainty and sensitivity analysis. Best practices in the development of ecosystem accounts will necessarily include the analysis and publication of uncertainties in order to enhance mutual learning, including the selection of the most appropriate modelling approaches to fill the accounts. The use of modelling techniques and approaches that allow uncertainties to be analyzed will be relevant in this context [44, 46, 49, 62].

### The policy relevance of capacity and related indicators

We believe the concept of capacity to also be directly relevant for key international and national policy initiatives. At a global policy level, sustainability discussions culminated in the adoption of the Sustainable Development Goals (SDGs) by the U.N. General Assembly in 2015. At least four out of 17 goals concern society’s connection to the environment. For example, SDG 2 (combat hunger) highlights the importance of sustainable land use for food production, SDG 12 focusses on the efficient use of natural resources to promote responsible production and consumption, and SDGs 14 and 15 focus on the protection, restoration and sustainable use of aquatic and terrestrial ecosystems, respectively. The concept of capacity encapsulates both the condition of environmental assets and the services derived from them. To gain insight in the sustainability of current ecosystem service supply and use, capacity-related indicators provide essential information to assess whether these policy targets can be met. Hence, the development of capacity measures across multiple ecosystem types and for a range of ecosystem services and data to monitor their trends over time would be a significant contribution to the measurement of progress toward the SDGs as well as various other linked environmental-economic policies.

At a European level, the Biodiversity Strategy 2020 [63] and the 7^th^ European Environmental Action Plan aim to halt the loss of biodiversity and ecosystem services and call on member states to develop natural capital accounts by 2020. Current design plans include the notion of ecosystem asset and capacity as possible indicators to be captured in the EU Ecosystem Accounts. In the U.S., recent guidance from the Executive Office of the President of the United States [64] point to an interest in more explicitly considering the effects of policies on ecosystem services. We believe that consideration of effects on both actual service flows and ecosystems’ capacity to supply services over the long term would be useful in each of these policy contexts, for instance by showing the difference between actual and sustainable ecosystem use in monetary terms.

Beyond the use of these concepts for natural capital accounting and other international and national-level goals, they also offer insights for local to regional scale land use and resource management planning. In each of our three case studies, the concepts we present offer a nuanced view of how future land use and resource management plans will impact ecosystems’ ability to sustainably provide services to people. Our case studies show, for example, (1) where it is possible to increase flows of one service without reducing flows of other services (Limburg carbon), (2) locations where use of provisioning services does and does not exceed rates of natural regeneration (Telemark timber harvest), and (3) where services are currently going unused or underused, and how increasing future use may undermine the landscape’s potential to provide services (Puget Sound viewsheds). Each example covers a single service, but all their underlying studies assessed multiple ecosystem services, enabling sophisticated tradeoff analyses. Comparing current flows of ecosystem services, sustainable flows under current management (capacity), and sustainable flows under alternative management (capability) permits an in-depth understanding of the implications of ecosystem change that can underpin a transition to sustainable ecosystem management. The three concepts can be expressed in both physical and monetary terms which can assist in selecting better ecosystem management approaches as well as in discussing the costs and benefits of different ecosystem management options. In addition the concepts permit a more comprehensive way of understanding and monitoring sustainability. Potential supply is a complementary indicator of sustainability–even when not currently in use, declines in ecosystems’ ability to provide services may be problematic for sustainability [17].

## Conclusions

This paper analyses how the concept of ecosystem asset can be understood, and highlights the relevance of the concept of capacity in frameworks aimed at understanding and conveying the importance of ecosystems. The concept of capacity speaks directly to questions of sustainable use of ecosystems whether in the context of the MA, IPBES, TEEB or accounting frameworks such as the SEEA EEA. This paper provides a conceptual framework for analysing the sustainability of ecosystem use by using an accounting lens that has a focus on the relationship between stocks and flows and capital and income. In particular, we define capacity and the related concepts of capability and potential supply, and illustrate how the three concepts can be quantified and used. The concepts are clearly related but their relevance for decision making will vary by analytical or policy context. An important advance that we contribute is in making the connection between measured changes in capacity and ecosystem degradation. This is a new and important step that is not considered in economic accounting since the standard view of assets as expressed in the SNA [21] does not account for their ability to renew themselves–an assumption that is appropriate when considering built or manufactured capital but not for natural capital. The capacity concept can convey information about the sustainability of ecosystem service supply that cannot be conveyed using standard approaches to degradation.

Importantly, we also demonstrate that the measurement of capacity and related concepts is feasible and not a purely theoretical exercise. However, much testing and discussion remains to refine and apply the concepts of ecosystem capacity, capability, and potential supply across a wide variety of ecosystem services and decision contexts. Key aspects for further research include understanding the links to intermediate services; agreeing on best solutions for integrating measures of capacity into accounting frameworks, including the measurement of degradation in accounts; developing a more wide spread understanding of the relationship between the condition of ecosystem assets and the services that they supply; and further applying these concepts toward ecosystem management. While further work remains, the concepts proposed here provide a basis for more consistent application and testing of these concepts in ecosystem accounting and ecosystem service assessment.
